# libRoadRunner 2.0: a high performance SBML simulation and analysis library

**DOI:** 10.1093/bioinformatics/btac770

**Published:** 2022-12-08

**Authors:** Ciaran Welsh, Jin Xu, Lucian Smith, Matthias König, Kiri Choi, Herbert M Sauro

**Affiliations:** Department of Bioengineering, University of Washington, Seattle, WA 98195, USA; Department of Bioengineering, University of Washington, Seattle, WA 98195, USA; Department of Bioengineering, University of Washington, Seattle, WA 98195, USA; Institute of Biology, Institute of Theoretical Biology, Humboldt-University Berlin, Berlin 10115, Germany; School of Computational Sciences, Korea Institute for Advanced Study, Seoul 02455, Republic of Korea; Department of Bioengineering, University of Washington, Seattle, WA 98195, USA

## Abstract

**Motivation:**

This article presents libRoadRunner 2.0, an extensible, high-performance, cross-platform, open-source software library for the simulation and analysis of models expressed using the systems biology markup language (SBML).

**Results:**

libRoadRunner is a self-contained library, able to run either as a component inside other tools via its C++, C and Python APIs, or interactively through its Python or Julia interface. libRoadRunner uses a custom just-in-time (JIT) compiler built on the widely used LLVM JIT compiler framework. It compiles SBML-specified models directly into native machine code for a large variety of processors, making it fast enough to simulate extremely large models or repeated runs in reasonable timeframes. libRoadRunner is flexible, supporting the bulk of the SBML specification (except for delay and non-linear algebraic equations) as well as several SBML extensions such as hierarchical composition and probability distributions. It offers multiple deterministic and stochastic integrators, as well as tools for steady-state, sensitivity, stability and structural analyses.

**Availability and implementation:**

libRoadRunner binary distributions for Windows, Mac OS and Linux, Julia and Python bindings, source code and documentation are all available at https://github.com/sys-bio/roadrunner, and Python bindings are also available via pip. The source code can be compiled for the supported systems as well as in principle any system supported by LLVM-13, such as ARM-based computers like the Raspberry Pi. The library is licensed under the Apache License Version 2.0.

## 1 Introduction

Dynamic network models (Sauro, 2014) of metabolic, gene regulatory, protein signaling and electrophysiological models require the specification of components, interactions, compartments and kinetic parameters. The systems biology markup language (SBML) (Hucka *et al.*, 2003; Keating *et al.*, 2020) has become the *de facto* standard for the declarative specification of these types of models (see SBML.org).

Popular tools for the development, simulation and analysis of models specified in SBML include COPASI (Hoops *et al.*, 2006), the systems biology workbench (SBW) (Bergmann and Sauro, 2006), the systems biology simulation core library (Panchiwala *et al.*, 2022), libSBMLSim (Takizawa *et al.*, 2013), iBioSim (Myers *et al.*, 2009), PySB (Lopez *et al.*, 2013), PySCeS (Olivier *et al.*, 2005) and VirtualCell (Moraru *et al.*, 2008), as well as many legacy tools that have been superseded by more modern software. Some of these applications are stand-alone packages designed for interactive use. Very few are reusable libraries. Currently, none are fast enough to support emerging applications that require large-scale simulation of network dynamics (Maggioli *et al.*, 2020). For example, multi-cell virtual-tissue simulations (Hester *et al.*, 2011) often require simultaneous simulation of tens of thousands of replicas of models residing in their cell objects and interacting between cells. In addition, optimization methods require the generation of time series for tens of thousands of replicas to explore the high-dimensional parameter spaces typical of biochemical networks (Bouteiller *et al.*, 2015).

Previously, we published libRoadRunner version 1, a cross-platform, multi-language library for fast execution of SBML model simulations. We designed libRoadRunner to provide: (i) Efficient time-series generation and analysis of large or multiple SBML-based models; (ii) A comprehensive and logical API; (iii) Interactive simulations in the style of IPython and MATLAB; and (iv) Extensibility. The library achieves its performance capabilities by compiling SBML directly into machine code ‘on-the-fly’ using LLVM as a ‘just-in-time’ (JIT) compiler (Lattner and Adve, 2004). The SBML model description is lexed and parsed into an abstract syntax tree using libSBML. Then libRoadRunner creates the necessary low-level LLVM intermediate representation (IR) code for compiling the SBML. Once compiled, the SBML representation of the model has been converted into an in-memory dynamic library from which symbols representing model functions can be exported and loaded into other languages. libRoadRunner wraps this low-level interface in user-friendly C and C++ APIs, which, in turn, provide the foundation for critical systems modelling tasks, such as model integration, steady-state analysis and metabolic control analysis (Somogyi *et al.*, 2015).libRoadRunner users usually fall into one of two categories: modellers or tool developers. Modellers use the libRoadRunner tool directly in their research for modelling dynamic systems (Karagöz *et al.*, 2021; Köller *et al.*, 2021a, b) or for developing new computational approaches such as detecting bistable switches (Reyes *et al.*, 2022) or performing dynamic flux balance analysis (Watanabe *et al.*, 2018). Tool developers, on the other hand, use libRoadRunner as a core SBML handling component in their modular software design, such as in Tellurium (Choi *et al.*, 2018), runBiosimulations (Shaikh *et al.*, 2021), MASSPy (Haiman *et al.*, 2021), SBMLsim (https://github.com/matthiaskoenig/sbmlsim), Compucell3D (Swat *et al.*, 2012), PhysiCell (Ghaffarizadeh *et al.*, 2018), pyBioNetFit (Neumann *et al.*, 2022), pyViPR (Ortega and Lopez, 2020) and DIVIPAC (Nguyen *et al.*, 2015).

**Fig. 1.**
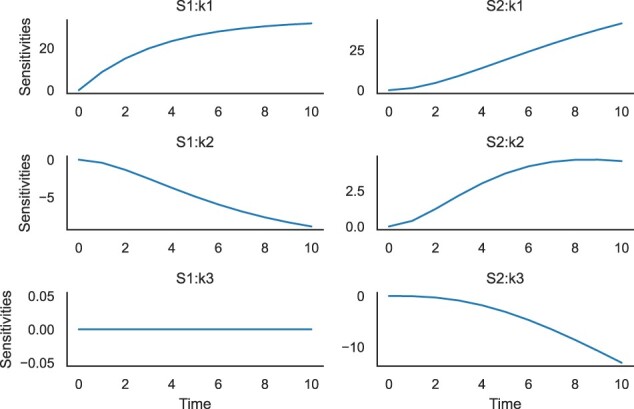
Time-dependent sensitivities for a simple linear chain model: X0 -> S1; k1*S1; S1 -> S2; k2*S2; S2 ->; k3*S2; k1 = 0.1; k2 = 0.3; k3 = 0.14; Xo = 10. Here, all possible combinations of the species (S1 and S2) and parameters (k1, k2 and k3) are shown. However, the interface allows us to be more selective if needed. The plots were generated by helper functions in libRoadRunner

In this work, we present libRoadRunner version 2. We have improved performance both for single-model and multi-model simulations. We have expanded the range of available features to include additional steady-state solvers, as well as time-dependent sensitivity analysis (see example in Fig. 1).

## 2 Major changes to 2.0

### 2.1 Performance improvements

In previous versions of libRoadRunner, loading many RoadRunner instances was slow because each model must JIT compile SBML to binary code. We have addressed this problem in several ways (i) by increasing the speed of compilation of a single model; (ii) by making it easy to compile multiple models simultaneously and (iii) by providing a ‘direct’ API for access to the model topology outside of modifying the SBML directly (reducing the amount of re-compilations).

#### 2.1.1 LLJit: a new JIT compiler

To increase the speed of compiling SBML to machine code, we have built a new JIT compiler called LLJit to replace the previous MCJit. LLJit uses LLVM version 13’s ‘ORC JIT v2’ API which provides an out-of-the-box but modular and customizable tool for JIT compiling LLVM IR code to machine code. It was not necessary to modify the LLVM IR generation stage of the compilation process, but a new strategy was designed to perform the compilation step. Our implementation of LLJit uses the standard object linking layer but a customized compile layer that automatically caches model object files in memory for fast reloading. The process of switching to the LLJit compiler is shown in Listing 1.

Listing 1 Python example of how to turn on the LLJit compiler. Variables: sbmlFile (str) is absolute path to sbml file on the disk.

from roadrunner import RoadRunner, Config

Config.setValue(

   Config.LLVM_BACKEND, Config.LLJIT)

rr = RoadRunner(sbmlFile)

#### 2.1.2 RoadRunnerMap: a parallel RoadRunner container

Because RoadRunner models are computationally expensive to compile, we have made it easy for users to make use of their multi-core system for compiling multiple models in parallel. libRoadRunner uses a lightweight abstraction around the standard C++17 threading library called thread_pool (Shoshany, 2021) for queuing build jobs and then storing references to compiled RoadRunner models in a thread-safe hash map structure called RoadRunnerMap. To construct a RoadRunnerMap, a collection of SBML files or strings are passed to the RoadRunnerMap constructor, along with an integer specifying the number of threads to use (Listing 2).

Listing 2 Python example of loading a list of SBML models in parallel using three threads. Variables: listOfSBML (List[str]) is a list of full paths to SBML files on the disk or strings in memory (or a mix thereof).

from roadrunner import RoadRunnerMap

rrm = RoadRunnerMap(listOfSBML, 3)

**Fig. 2.**
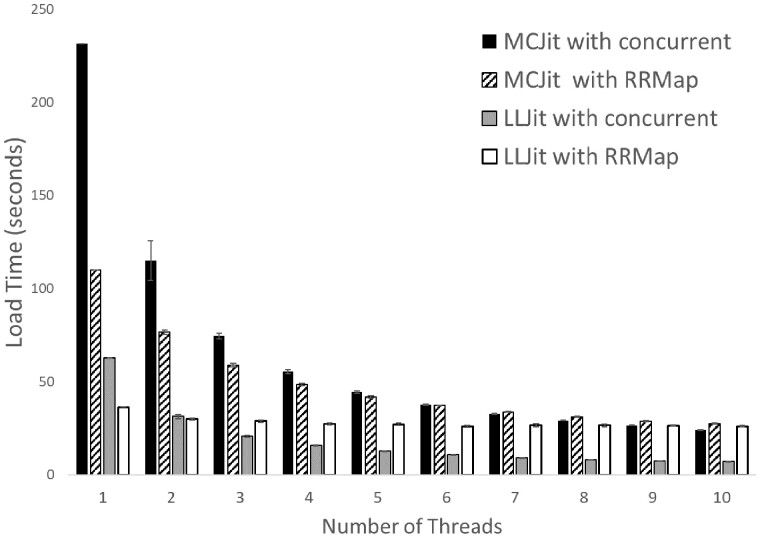
Time to load over 1100 models from the BioModels database with libRoadRunner. Data are included for the two different JIT backends (MCJit and LLJit), using the RoadRunnerMap construct or not, and using the given number of threads. Shown is the average of 10 replicates with their standard deviations. Not shown: when increasing the number of threads to 23, the total times were only reduced by a maximum of an additional 2%

To demonstrate the capabilities of libRoadRunner v2, we have measured the time it takes for the MCJit and LLJit compilers to load and simulate over 1100 models from the curated section of the BioModels database (Malik-Sheriff *et al.*, 2020) using either the native-Python concurrent package, or using the RoadRunnerMap construct, both with different numbers of threads. As can be seen in Figure 2, the new LLJit compiler is more than three times faster than the previous MCJit compiler in both contexts, making it the fastest compiler we have built to date. Increasing the number of threads decreases runtime but with diminishing returns. The RoadRunnerMap construct was seen to be faster than the native Python ‘concurrent’ multithreading approach at lower numbers of threads, but slower than the native at higher numbers of threads. We hypothesize this is due to the RoadRunnerMap controlling thread taking up a significant portion of the clock time: because the parallelization occurs entirely in C++, the Python thread itself remains unoptimized. When using the native-Python concurrent package, that control is better managed by Python itself.

#### 2.1.3 Pickled (serialized) RoadRunner

Once loaded, users can save a model’s state either to a binary string for in-memory storage or to a disk for persistent storage. The result is a platform-specific binary snapshot of a RoadRunner object which can be reloaded with significant performance improvements compared to recompiling the model. Listing 3 demonstrates this functionality.

Listing 3 Example of saving a RoadRunner object’s state to a file and then loading it again. Variables: sbmlFile (str) is a full path to a valid SBML file on the disk; fileName (str) is a full path to where to save the model state on the disk.

from roadrunner import RoadRunner

rr = RoadRunner(sbmlFile)

*# save state to string*

rr.saveState(fileName)

*# load state*

rrReloaded = RoadRunner()

rrReloaded.loadState(fileName)

In Python, a prerequisite for using RoadRunner with various parallel or multithreading toolboxes is the ability to serialize an instance of a RoadRunner object using Python’s standardized ‘pickle’ protocol. We have built an adaptor between our in-house RoadRunner serialization strategy and Python’s pickle protocol so that our users can now build their own parallel applications on top of libRoadRunner. We anticipate that this will be valuable to the systems biology community, particularly for problems involving repeated time-series simulations such as optimization or stochastic simulations. An example of the latter is shown in Listing 4.

Listing 4 Example of using RoadRunner object with Python’s multiprocessing library to stochastically simulate a model hundred thousand times.

from multiprocessing import Pool

from roadrunner import RoadRunner

def simulate_worker(rr: RoadRunner):

    rr.resetAll()

    return rr.simulate(0, 10, 11)

rr = RoadRunner(sbmlFile)

rr.setIntegrator(’gillespie’)

if __name__==’__main__’:

    p = Pool(processes = 8)

    results = p.map(

      simulate_worker,

      [rr for i in range(100000)])

### 2.2 Direct API

In libRoadRunner version 1, any changes to the model structure required the modification, re-parsing and re-compiling of the SBML. Since these operations are computationally expensive and potentially convoluted, we have implemented an API for interacting directly with the internal object model. This ‘direct’ API allows users to add and remove SBML components such as compartments, species, reactions and events programmatically, without the need to re-parse the model after each change. Since these changes require model recompilation before use, we provide an argument called forceRegenerate to all direct API functions which give users the ability to control when the model is recompiled—i.e. only after all model changes are complete. Listing 5 provides an example using this API.

Listing 5 Example of adding a simple first-order mass action degradation reaction to a loaded SBML model. The code assumes that a compartment called “cell” was loaded in the initial sbmlFile. Variables: sbmlFile (str) is a full path to a valid SBML file on the disk.

from roadrunner import RoadRunner

rr = RoadRunner(sbmlFile)

rr.addSpecies(“A”, “cell”,

     initConcentration = 5.0,

     forceRegenerate=False

)

rr.addReaction(“ADeg”, [“A”], [], 0.5*A, True)

### 2.3 Julia language bindings

The Julia programming language has gained traction with the systems biology community in recent years. We have therefore implemented language bindings to connect Julia users to libRoadRunner. While our Python bindings are implemented using SWIG (Beazley *et al.*, 1996), there is no Julia interface for SWIG, so our Julia bindings use ccall internally to expose symbols from the libRoadRunner shared library to a Julia API. Listing 6 demonstrates the use of this API in Julia.

Listing 6 An example showing how to load an SBML model and perform a simulation in Julia. The first two lines install the libRoadRunner language bindings and the rest of the code compiles an SBML model sbmlString and runs a simulation using the simulateEx method.

*# get julia bindings*

import Pkg

Pkg.add(“RoadRunner”)

*# simulate a model*

using RoadRunner

rr = RoadRunner.createRRInstance()

RoadRunner.loadSBML(rr, sbmlString)

S = RoadRunner.getFloatingSpeciesIds(rr)

data = RoadRunner.simulateEx(rr, 0, 40, 500)

### 2.4 Plugin system

We have developed a flexible and robust plugin system that tightly integrates extensions to the libRoadRunner with various functionalities. Anyone can incorporate a computational routine based on an instance of libRoadRunner which can greatly increase the performance, with the only requirement being the plugin code to be written in C++ and available at the compile time. Several examples such as parameter estimation algorithms that use libRoadRunner solvers and an interface to AUTO (Doedel, 1981) for bifurcation analysis are available in the documentation. Listing 7 illustrates a simple example of using the AUTO plugin for bifurcation analysis.

Listing 7 An example showing how to load a model into AUTO plugin, set parameters, run bifurcation analysis, and plot the bifurcation diagram.

from rrplugins import *

auto = Plugin(“tel_auto2000”)

 

*# Set parameters*

auto.setProperty(“SBML”, sbmlString)

auto.setProperty(“NMX”, 5000)

auto.setProperty(“ScanDirection”, “Positive”)

 

*# Execute the plugin*

auto.execute()

 

*# Plot bifurcation diagram*

pts = auto. BifurcationPoints

lbls = auto. BifurcationLabels

biData = auto. BifurcationData

biData.plotBifurcationDiagram(pts, lbls)

### 2.5 Miscellaneous new functionality

In 2019, a new SBML specification was released [level 3 version 2 (Hucka *et al.*, 2019)]. We now support the features of the new specification, including additional MathML functions, cases with ‘missing’ elements that are now valid in SBML, and the presence of Boolean values where numeric values are expected, and vice versa. In addition, we now fully support the ‘Distributions’ SBML package (Smith *et al.*, 2020), which defines new functions that stochastically draw values from known distributions, even in otherwise deterministic conditions (such as initial species levels).

Version 2 of libRoadRunner also includes a number of other miscellaneous changes. These include additions to numerical routines used to solve differential equations for the time course and for computing the steady state. In particular, we have implemented a basic Euler integration method which has been used for certain time-critical applications and the RK45 solver which can be used to double-check the accuracy of the time course solution generated by the default CVODE implementation. We have also included support for variable stoichiometries in reactions. The Antimony language (Smith *et al.*, 2009) has been updated to reflect this change.

Like our CVODE implementation, our time series sensitivity implementation uses the popular Sundials package (Hindmarsh *et al.*, 2005). Specifically, we have two strategies for solving the sensitivity equations. They can either be solved simultaneously with the system equations (Maly and Petzold, 1996) or solved using a staggered approach (Caracotsios and Stewart, 1995).libRoadRunner version 2 also makes use of the Sundials ‘kinsol’ library for new steady-state solvers. Specifically, we support the Inexact Newton approach (Brown, 1987). As a result, version 2 now gives the user access to two nonlinear solvers.

For Windows users, we also provided an updated installer that will install an independent but complete working environment for biochemical modelling. The installer distributes the latest Spyder IDE (https://www.spyder-ide.org/) as well as the Jupyter notebook interface (Kluyver *et al.*, 2016). Note that libRoadRunner and associated tools can be easily installed on open platforms such as Colaboratory (Carneiro *et al.*, 2018) using pip.

User control over roadrunner functionality has also been improved. Time-series simulations, steady-state calculations and approximation routines, among others, now have more options for the user and can be used on a wider variety of models. Other changes in libRoadRunner version 2 include improved compliance with the SBML Test Suite (Smith, 2022), and a new automatic build and test system using updated dependencies, which allows us to release more frequently, with fewer bugs. Overall, we have resolved over 250 issues since 2018, filed by 23 different people.

## 3 Discussion

libRoadRunner is a fast and convenient tool for both individuals who are investigating the dynamics of a biological system and tool developers who are building new methods for solving and analysing such systems. In version 2, we have built a variety of new tools for the construction, compilation, analysis and solving of dynamical systems described in SBML.

libRoadRunner version 1 was highly optimized for the simulation of dynamical systems thanks to our JIT compilation strategy. As a result, libRoadRunner was the fastest available SBML dedicated simulator (Maggioli *et al.*, 2020; Panchiwala *et al.*, 2022; Somogyi *et al.*, 2015). However, one of the disadvantages of our strategy is that when the need arises for the simulation of many SBML models together, the run time is dominated by the compile time. Examples of such a need include ensemble modelling, where many instances of SBML with varying parameters or typologies need to be simulated simultaneously. With the new changes we describe in this article, our performance metrics have increased even further.

To alleviate this bottleneck, we have prioritized new features that enhance the speed with which a model can be compiled. One such feature is an entirely new compiler called LLJit which sits side-by-side with the older MCJit. We have demonstrated that LLJit is significantly faster than earlier libRoadRunner implementations (Fig. 2) at compiling the same code. Similarly, once loaded, a model may be modified (even to the extent of adding or removing model elements) more rapidly than loading a new model from scratch, and new functions have been added to allow this, as well.

While decreasing the compile time is a worthy goal, there is a natural limit to the speed with which a single model can be compiled. An alternative mechanism for enhancing performance in multi-model problems is to make better use of the available resources that exist in most modern computer systems using parallelism. We have introduced parallelism in two ways. First, we have built a RoadRunner container called RoadRunnerMap which is capable of orchestrating parallel compiles. Secondly, we have implemented support for Python’s pickle protocol. While the former enables us to abstract parallelism away from the user completely, the latter allows our more experienced users to devise their own parallel computation.

## 4 Conclusion

With the advent of the changes introduced into libRoadRunner version 2, the library is now more efficient in running, loading and changing models at runtime. These features were added to support a number of specific use cases. These include two main applications: parameter optimization on large compute clusters, and using libRoadRunner to create large model ensembles that include variation in parameters as well as rate laws and network topology.

## Data Availability

libRoadRunner source code is available from our GitHub webpage at https://github.com/sys-bio/roadrunner/ and precompiled binaries are available for Windows, Mac OS and Linux operating systems at https://github.com/sys-bio/roadrunner/releases. The Windows front end for Tellurium (which includes the latest version of roadrunner) is available at https://github.com/sys-bio/tellurium#front-end-1-tellurium-spyder-ide. Python bindings are available both as wheel files from the releases page and from pypi.org installable through pip: pip install libroadrunner The Julia source code is available at https://github.com/sys-bio/RoadRunner.jl and can be installed via Julia’s package manager with Pkg.add(”RoadRunner”). The scripts used to generate the data for this article are available at https://github.com/saurolab-papers/rr2.0_paper.
